# Predictive risk factors of complications in different breast reconstruction methods

**DOI:** 10.1007/s10549-020-05705-3

**Published:** 2020-05-28

**Authors:** J. S. Palve, T. H. Luukkaala, M. T. Kääriäinen

**Affiliations:** 1 Department of Plastic Surgery, Faculty of Medicine and Health Technology and Tampere University Hospital, Tampere University, Elämänaukio 2, 33521 Tampere, Finland; 2 Research, Development and Innovation Center, Faculty of Social Sciences, Tampere University Hospital and Health Sciences, Tampere University, Tampere, Finland

**Keywords:** Breast reconstruction, Complication, Risk factor, DIEP, LD, Implant reconstruction

## Abstract

**Purpose:**

Women with different BMI, age and comorbidities seek for breast reconstruction. It is critical to understand the risk associated with each technique to ensure the most appropriate method and timing is used. Outcome after reconstructions have been studied, but consensus is lacking regarding predictive risk factors of complications. The authors present their experience of different autologous and alloplastic reconstructions with an emphasis on predictors of complications.

**Methods:**

Prospectively maintained reconstruction database from 2008 to 2019 was reviewed. Factors associated with complications were identified using logistic regression, multinomial logistic regression and risk factor score to determine predictors of complications.

**Results:**

A total of 850 breast reconstructions were performed in 793 women, including 447 DIEP, 283 LD, 12 TMG and 51 implant reconstructions. Complications included minor (*n* = 231, 29%), re-surgery requiring (*n* = 142, 18%) and medical complications (*n* = 7, 1%). Multivariable analysis showed that complications were associated independently with BMI > 30 (OR 1.59; 95% CI 1.05–2.39, *p* = 0.027), LD technique (OR 4.05; 95% CI 2.10–7.81, *p* < 0.001), asthma or chronic obstructive pulmonary disease (OR 2.77; 95% CI 1.50–5.12, *p* = 0.001) and immediate operation (OR 0.69; 95% CI 0.44–1.07, *p* = 0.099). Each factor contributed 1 point in the creation of a risk-scoring system. The overall complication rate was increased as the risk score increased (35%, 61%, 76% and 100% for 1, 2, 3 and 4 risk scores, respectively, *p* < 0.001).

**Conclusions:**

The rate of complication can be predicted by a risk-scoring system. In increasing trend of patients with medical problems undergoing breast reconstruction, tailoring of preventive measures to patients’ risk factors and careful consideration of the best timing of reconstruction is mandatory to prevent complications and costs.

## Introduction

Although breast conservation therapy is an effective option for patients with early stage breast cancer, many women still undergo mastectomy for either cancer treatment or prophylaxis [1]. Increasing use of mastectomy also in early breast cancer has been reported, along with increasing use of bilateral mastectomy, even for unilateral disease [2]. Women who undergo mastectomy may perceive a negative self-image and experience negative changes in their sexuality [3]. Breast reconstruction following either prophylactic or therapeutic mastectomy may increase the quality of life [1]. Breast reconstructions can be performed either immediately or delayed and can be divided into implant-based, autologous tissue and combined reconstructions [4]. Immediate breast reconstruction is often recommended for women undergoing mastectomy because it is thought to confer psychosocial benefits and result in better cosmesis [2]. However, for medical or oncological reasons, some patients may be poor candidate for immediate reconstruction [1]. Immediate reconstructions are also associated with higher rates of complications compared to delayed reconstructions [1, 2].

Women with different habitus, BMI, age and comorbidities seek for breast reconstruction. With numerous reconstructive options available, it is critical to understand the risk associated with each reconstructive technique to ensure that the most appropriate method is used. Every breast reconstruction method is associated with its own surgical complication profile and its own impact on quality of life [5]. It has been concluded that women with autologous reconstruction were significantly more satisfied with their reconstructed breasts than women with alloplastic reconstruction [4, 5]. Satisfaction with outcome and quality of life after reconstructions have been widely studied [1, 3–7], but consensus is lacking in the literature regarding differences in complication rates after different reconstructions [2]. Data are available concerning surgical site infection and noninfectious complications after breast reconstruction in different techniques. Commonly, studies have evaluated a single method or have compared two different methods [8]. Usually, certain comorbid conditions and postoperative complications have been assessed but studies are limited regarding predictive risk factors of complications.

## Aim of the study

The aim of this study was to determine complications graded with Clavien–Dindo classification identified within 90 days of operation in autologous and alloplastic reconstruction techniques both in delayed and immediate procedures. We also aimed to determine predictive risk factors of complications in different breast reconstruction techniques to decrease complications and burden of health care costs and facilitate discussion of options with women considering breast reconstruction.

## Patients and methods

This retrospective study was conducted using data from Tampere university hospital (Finland) breast reconstruction database. We identified all performed breast reconstruction operations from January 1, 2008, through December 31, 2019. Permission to access the clinical records of the patients for the study was obtained from the scientific center of Tampere University Hospital. By reviewing the clinical records, we ensured that there were no duplicates.

We collected data on patient characteristics, reconstruction indication, technique and complications. The definitions of collected data are presented in Table 1. In our hospital, five different reconstruction methods have mainly be used: (1) deep inferior epigastric perforator flap (DIEP) (with or without lymph node transfer), (2) latissimus dorsi (LD) flap (either plain, with immediate lipofilling or with implant), (3) tissue expander with a secondary silicone implant, (4) direct implant augmentation and (5) transverse musculocutaneous gracilis flap (TMG). The same surgeons performed all types of reconstructions. The primary outcome measure was complications. All postoperative complications were scored using Clavien–Dindo classification (Table 1). In this classification, a complication is defined as any deviation from the normal postoperative course [9]. The number of complications per patient was scored. The time frame for identifying complications was 90 days after reconstructive operation.

**Table 1:** Definitions of collected data

	Definition
Patient characteristics	Age Body mass index (BMI) in kg/m^2^ Smoking status Smoker: active smoker or patient who stopped smoking for a period of 4 weeks prior the reconstruction Non-smoker: patient who had never smoked Comorbidities Diabetes Cardiovascular disease (CDV) Asthma or chronic obstructive pulmonary disease (COPD) Hypothyreosis Other
Adjuvant therapies	Radiotherapy before or after reconstruction Adjuvant chemotherapy Adjuvant hormonal therapy Intake of Tamoxifen was ceased 4 weeks prior the operation
Reconstruction indication	Delayed Immediate (including prophylactic procedures)
Reconstruction technique	DIEP (deep inferior epigastric perforator flap) With or without lymph node transfer LD (latissimus dorsi) Plain With immediate lipofilling With implant Implant Tissue expander with a secondary silicone implant Direct implant augmentation TMG (transverse musculocutaneous gracilis flap)
Complications (scored according to Clavien–Dindo classification)	Grade I: seroma, other minor deviation from normal postoperative course without the need for pharmacological treatment or surgical interventions Grade II: infection without surgical intervention, but requiring per oral pharmacological treatment (antibiotics) Grade III: deep infection, hematoma, skin or fat necrosis requiring surgical intervention in operation theater Grade IV: life-threatening complication (e.g., pulmonary embolism)

Grade I and II were defined as minor complications, grade III as re-surgery requiring complication and grade IV as medical complication

The datasets analyzed during the current study are available from the corresponding author on reasonable request.

## Statistics

Differences between reconstruction techniques (Tables 2 and 4) were tested using Pearson Chi-Square test or Fisher’s Exact test. Univariable and multivariable logistic regression analyses were applied to estimate odds ratios (ORs) and 95% confidence intervals (CIs) to analyze the difference between immediate and delayed reconstructions (Table 3) and multivariable association between possible risk factors for complications (Table 6). Multinomial logistic regression was used to compare different complication types to cases without complication (Table 5) showing results by ORs with 95% CIs. Risk-scoring system to stratify risk for postoperative complication was developed by using significant variables (BMI > 30, LD, asthma/COPD and immediate indication) from the multivariable logistic regression analysis. Each factor contributed 1 point. Risk score was defined as the sum of the presence for each component. A *p* value < 0.05 was considered statistically significant. IBM SPSS Statistics version 26.0 for Windows software (SPSS Inc. Chicago, Illinois) was used for statistical analyses.

**Table 2:** Summary of demographic parameters for the whole group and for each reconstruction technique (*N* = 793)

	Whole group (*n* = 793)		Implant (*n* = 51)		LD (*n* = 283)		Microvascular (*n* = 459)		*p* value
	*n*	(%)	*n*	(%)	*n*	(%)	*n*	(%)	
Age									< 0.001
< 45 years	147	(18)	16	(31)	41	(14)	90	(20)	
45–65 years	594	(75)	28	(55)	211	(75)	355	(77)	
> 65 years	52	(7)	7	(14)	31	(11)	14	(3)	
BMI									< 0.001
< 25	313	(39)	38	(74)	120	(43)	155	(34)	
25–30	340	(43)	9	(18)	100	(35)	231	(50)	
> 30	140	(18)	4	(8)	63	(22)	73	(16)	
Smoking									0.287
No	718	(91)	45	(88)	251	(89)	422	(92)	
Yes	75	(9)	6	(12)	32	(11)	37	(8)	
Comorbidities									
Cardio vascular disease									0.002
No	651	(82)	45	(88)	214	(76)	392	(85)	
Yes	142	(18)	6	(12)	69	(24)	67	(15)	
Diabetes									0.555
No	769	(97)	50	(98)	272	(96)	447	(97)	
Yes	24	(3)	1	(2)	11	(4)	12	(3)	
Hypothyreosis									0.512
No	711	(90)	46	(90)	249	(88)	416	(91)	
Yes	82	(10)	5	(10)	34	(12)	43	(9)	
Asthma/COPD									0.981
No	736	(93)	47	(92)	263	(93)	426	(93)	
Yes	57	(7)	4	(8)	20	(7)	33	(7)	
None of above									0.002
No	260	(33)	15	(29)	115	(41)	130	(28)	
Yes	533	(67)	36	(71)	168	(59)	329	(72)	
Radiation therapy									0.001
Yes	419	(53)	15	(29)	143	(51)	261	(57)	
No	374	(47)	36	(71)	140	(49)	198	(43)	
Tamoxifen									0.018
Yes	316	(40)	22	(43)	94	(33)	259	(56)	
No	477	(60)	29	(57)	189	(67)	200	(44)	
Indication									< 0.001
Delayed	672	(85)	26	(51)	241	(85)	405	(88)	
Immediate	121	(15)	25	(49)	42	(15)	54	(12)	
Complication									< 0.001
None	413	(52)	31	(61)	84	(30)	298	(65)	
I	179	(23)	6	(12)	142	(50)	31	(7)	
II	52	(7)	3	(6)	15	(5)	34	(7)	
III	142	(18)	9	(18)	42	(15)	91	(20)	
IV	7	(1)	2	(4)	0	(0)	5	(1)	
Number of complications									< 0.001
0	412	(52)	31	(61)	83	(29)	298	(65)	
1	287	(36)	13	(25)	160	(57)	114	(25)	
2	80	(10)	6	(12)	36	(13)	38	(8)	
3	14	(2)	1	(2)	4	(1)	9	(2)	
Location of complication									< 0.001
None	416	(53)	31	(61)	84	(30)	301	(65)	
Flap	121	(15)	20	(39)	11	(4)	90	(20)	
Donor site	212	(27)	0	(0)	163	(58)	49	(11)	
Both	44	(5)	0	(0)	25	(8)	19	(4)

**Table 3:** Characteristics of patients and complications in different breast reconstruction techniques in delayed and immediate reconstructions (*N* = 793)

	Delayed		Immediate		Immediate vs. delayed			
	(*n* = 672)		(*n* = 121)		Crude		Multivariable	
	*n*	(%)	*n*	(%)	OR	(95% CI)	OR	(95% CI)
Age								
< 45 years	112	(17)	35	(29)	1.00		1.00	
45–65 years	516	(77)	78	(64)	0.48	(0.31–0.76)	0.51	(0.31–0.85)
> 65 years	44	(7)	8	(7)	0.58	(0.25–1.35)	0.49	(0.19–1.29)
BMI								
≤ 25	253	(38)	60	(50)	1.00		1.00	
25–30	299	(44)	41	(34)	0.58	(0.38–0.89)	0.87	(0.54–1.41)
≥ 30	120	(18)	20	(16)	0.70	(0.40–1.22)	1.04	(0.56–1.91)
Smoking								
No	611	(91)	107	(88)	1.00		1.00	
Yes	61	(9)	14	(12)	1.31	(0.71–2.43)	1.29	(0.67–2.47)
Comorbidities								
CVD	127	(19)	15	(12)	0.61	(0.34–1.08)	0.95	(0.32–2.81)
DM	21	(3)	3	(2)	0.79	(0.23–2.68)	1.22	(0.31–4.84)
Asthma/COPD	46	(7)	11	(9)	1.36	(0.68–2.71)	2.29	(0.80–6.61)
Hypothyreosis	69	(10)	13	(11)	1.05	(0.56–1.97)	1.63	(0.51–5.26)
None	447	(67)	86	(71)	1.24	(0.81–1.89)	1.75	(0.53–5.78)
Radiation therapy								
No	290	(43)	84	(69)	1.00		1.00	
Yes	382	(57)	37	(31)	0.33	(0.33–0.51)	0.35	(0.22–0.54)
Reconstruction technique								
Microvascular	405	(60)	54	(45)	1.00		1.00	
LD	241	(36)	42	(35)	1.31	(0.85–2.02)	1.32	(0.78–2.23)
Implant	26	(4)	25	(21)	7.21	(3.89–13.4)	5.62	(2.86–11.1)
Complication								
None	358	(53)	55	(46)	1.00		1.00	
I	153	(23)	26	(21)	1.11	(0.67–1.83)	1.16	(0.63–2.14)
II	45	(7)	7	(6)	1.01	(0.43–2.36)	1.14	(0.46–2.82)
III	111	(16)	31	(26)	1.82	(1.12–2.96)	1.94	(1.14–3.30)
IV	5	(1)	2	(2)	2.60	(0.49–13.8)	1.79	(0.27–11.1)

## Results

A total of 850 breast reconstructions were performed in 793 women during the study period. Of these patients, 672 (85%) had delayed reconstruction and 121 (15%) immediate reconstruction. Bilateral reconstruction was performed in 57 (7%) patients. DIEP reconstruction was the most common reconstruction method (56%, *n* = 447), followed by LD (36%, *n* = 283), implant (6%, *n* = 51) and TMG reconstruction (2%, *n* = 12). LD reconstruction included 132 LD flaps ± immediate lipofilling and 151 flaps with implant. Diep reconstructions included 36 DIEPs with lymph node transfer.

The summary of demographic parameters for the whole group and for each reconstruction technique is presented in Table 2. A complication was recorded in 380 (48%) of 793 patients. The most common complication was seroma (179/793, 23%), followed by complication requiring surgical intervention (142, 18%), superficial infection treated with antibiotics (52, 7%) and life -threatening complication (7, 1%). All life-threatening complications recorded in this study were pulmonary embolisms. No deaths were recorded. Less complications were associated with microvascular reconstructions, because 298 (65%) of 459 patients recovered without any complication compared to implant reconstructions (61%) and LD reconstructions (30%). There were no significant differences in complications in LDs with or without implant (*p* = 0.863). Complications occurred more commonly on flap donor site (212/380, 56%) than in reconstructed breast (121/380, 32%). Implant reconstructions had most commonly complications in reconstructed breast area (39%) compared to microvascular flaps (20%) and LD reconstructions (4%). LD reconstruction patients had more commonly complications in the donor site (58%) than patients with microvascular flap reconstruction (11%).

Immediate reconstructions had complications more commonly than delayed, especially complications requiring re-surgery were more common (26% vs. 16%, multivariable OR 1.94, 95% CI 1.14–3.30). Detailed characteristics of patients and complications in different reconstruction techniques in immediate and delayed reconstructions are presented in Table 3.

Patients with LD reconstructions had more commonly overweight (22% BMI > 30), while in implant reconstruction group 74% were normal weighted (BMI < 25). Reconstruction was most common at the age of 45–65 years in all reconstruction groups. Comorbidities were recorded in 33% (257/793) of patients. LD patients had more comorbidities (41%) compared to implant (29%) or microvascular (28%) groups. Cardiovascular disease was the most common comorbidity (131/257, 51%), followed by hypothyreosis (82/257, 32%), asthma or COPD (57/257, 22%) and diabetes (24, 9%). Microvascular and implant reconstruction patients had more commonly antiestrogen medication (43–44%) compared to LD group (33%).

Radiation therapy was performed to 53% (419/793) patients. Of these patients, 9% (37/419) received it postoperatively. Of the patients, 75 (9%) were smokers. All smokers had ceased smoking for 4 weeks preoperatively. The amount of smokers was highest in implant (12%) and LD reconstruction (11%) groups compared to microvascular reconstructions (8%).

We also evaluated in detail the complications requiring surgical intervention (Table 4). Postoperative hematoma was most common in LD flaps (most commonly in donor site), while skin or fat necrosis in reconstructed breast was most common in DIEP flaps. The revision of microvascular anastomosis was required in 22 cases (4.8%). Total flap loss was six (1%) in microvascular flaps and 0 in LD flaps.

**Table 4:** Detailed analysis of complications requiring re-surgery (Clavien–Dindo III) in different reconstruction techniques (*N* = 142)

	Implant (*N* = 51)		LD (*N* = 283)		Microvascular (*N* = 459)	
	*n*	(%)	*n*	(%)	*n*	(%)
Hematoma	3	(5.9)	22	(7.8)	17	(3.7)
Skin or fat necrosis	4	(7.8)	12	(4.2)	40	(8.7)
Deep infection	2	(3.9)	8	(2.8)	12	(2.6)
Revision of vascular anastomosis	0		0		22	(4.8)
Total	9	(17.6)	42	(14.8)	91	(19.8)

Differences between reconstruction techniques (*p* < 0.001) were tested using Fisher’s exact test

Total flap loss was six (6/459, 1%) in microvascular flaps and 0 in LD flaps

Multivariable analyses were performed to identify risk factors for postoperative complications. The OR and 95% CI of each potential risk factor were estimated. In multivariable analysis, we included clinical variables with surgical variables including indication and reconstruction technique (Tables 5, 6). BMI > 30, LD reconstruction technique, immediate reconstruction and asthma/COPD were significantly associated with postoperative complications after breast reconstruction. We calculated risk factor scores by adding the number of significant fore mentioned risk factors in the multivariable logistic regression analysis. Each factor contributed 1 point in the creation of a risk-scoring system. The overall complication rate was increased as the risk score increased (35%, 61%, 76% and 100% for 1, 2, 3 and 4 risk scores, respectively, *p* < 0.001) (Table 7).

**Table 5:** Multivariable predictive risk factor analysis

	I (*n* = 179)			II (*n* = 52)				III (*n* = 142)			IV (*n* = 7)		
	*n*	OR	(95% CI)	*n*	OR	(95% CI)	*n*		OR	(95% CI)	*n*	OR	(95% CI)
Age													
< 45	25	1.00		10	1.00		31		1.00		1	1.00	
45–65	132	1.15	(0.64–2.05)	39	0.85	(0.39–1.82)	106		0.80	(0.49–1.31)	6	2.06	(0.22–18.9)
> 65	22	1.67	(0.69–3.99)	3	0.79	(0.19–3.28)	5		0.47	(0.16–1.39)	0	–	(–)
BMI													
≤ 30	143	1.00		40	1.00		108		1.00		5	1.00	
> 30	36	1.23	(0.71–2.12)	12	1.65	(0.79–3.46)	34		1.87	(1.13–3.09)	2	3.55	(0.60–21.1)
Cardiovascular disease													
No	138	1.00		41	1.00		117		1.00		6	1.00	
Yes	41	0.95	(0.55–1.62)	11	1.16	(0.53–2.53)	25		0.98	(0.56–1.70)	1	0.72	(0.07–7.20)
Diabetes mellitus													
No	173	1.00		50	1.00		135		1.00		7	1.00	
Yes	6	1.29	(0.39–4.29)	2	1.55	(0.31–7.61)	7		2.14	(0.75–6.09)	0	–	(–)
Asthma or COPD													
No	162	1.00		44	1.00		129		1.00		6	1.00	
Yes	17	2.79	(1.25–6.20)	8	3.96	(1.59–9.86)	13		2.30	(1.07–4.93)	1	3.17	(0.31–32.0)
Hypothyreosis													
No	162	1.00		45	1.00		122		1.00		7	1.00	
Yes	17	0.81	(0.40–1.61)	7	1.48	(0.61–3.58)	20		1.53	(0.84–2.77)	0	–	(–)
Radiation therapy													
No	85	1.00		20	1.00		70		1.00		4	1.00	
Yes	94	1.14	(0.75–1.72)	32	1.42	(0.77–2.63)	72		0.98	(0.65–1.46)	3	0.79	(0.16–3.96)
Indication													
Delayed	153	1.12	(0.61–2.05)	45	1.13	(0.46–2.78)	111		1.87	(1.10–3.17)	5	1.84	(0.26–12.8)
Immediate	26	1.00		7	1.00		31		1.00		2	1.00	
Reconstruction technique													
Implant	6	1.80	(0.66–4.88)	3	0.93	(0.25–3.41)	9		0.82	(0.36–1.86)	2	4.30	(0.61–30.5)
LD	142	16.2	(10.2–26.0)	15	1.58	(0.81–3.10)	42		1.58	(1.00–2.48)	0	–	(–)
Microvascular	31	1.00		34	1.00		91		1.00		5	1.00	

Multinomial logistic regression analysis was used showing results by odds ratios (OR) with 95% confidence intervals (CI) for seroma (I), superficial infection (II), re-surgery requiring complication (III) and pulmonary embolism (IV) using none complication as reference group (*n* = 413), Total *N* = 793

**Table 6:** Risk factors for complications (*n* = 380, 47.9%)

	Complication		
	OR	(95% CI)	*p*
Age years			0.93
< 45	1.00		
45–65	0.93	(0.62–1.38)	0.71
> 65	0.97	(0.48–1.97)	0.94
BMI			
BMI ≤ 30	1.00		
BMI > 30	1.59	(1.05–2.39)	0.027
Smoking			
No	1.00		
Yes	1.45	(0.86–2.43)	0.16
Radiation therapy			
No	1.00		
Yes	1.10	(0.80–1.50)	0.56
Reconstruction			< 0.001
Implant	1.00		
LD	4.05	(2.10–7.81)	< 0.001
Microvascular	0.93	(0.49–1.75)	0.82
DM	1.63	(0.65–4.08)	0.29
CVD	0.99	(0.65–1.50)	0.96
Asthma or COPD	2.77	(1.50–5.12)	0.001
Hypothyreosis	1.17	(0.71–1.92)	0.54
Indication			
Delayed	1.00		
Immediate	0.69	(0.44–1.07)	0.099

**Table 7:** Rate of complications according to risk factor score (0–4), including BMI > 30, asthma/COPD, immediate indication and LD as a reconstruction technique

		Risk sum score					*p*
		0	1	2	3	4	
Number of the patients, *n* (% of all patients)		65 (8.2)	382 (48.2)	270 (34.0)	74 (9.3)	2 (0.3)	
Overall complications, *n* (% of risk sum scores)							< 0.001
Seroma	4 (2.2)	38 (21)	102 (57)	34 (19)	1 (0.6)		
Superficial infection	3 (5.8)	26 (50)	15 (29)	8 (15)	0 (0)		
Surgery demanding complication	18 (13)	63 (44)	47 (33)	13 (9.2)	1 (0.7)		
Pulmonary embolism	1 (14)	5 (71)	0 (0)	1 (14)	0 (0)		
Total complications *n* (% of patients)	26 (40)	132 (35)	164 (61)	56 (76)	2 (100)		

Differences between risk factor sum scores were tested by Fisher’s exact test

## Discussion

We evaluated the preoperative clinical and surgical variables for the different breast reconstruction techniques in 793 reconstruction patients of our clinical database to predict postoperative complications after operation. We found that BMI > 30, asthma/COPD, LD technique and immediate reconstruction were significantly associated with postoperative complications. By using these four variables, we stratified risk scores for postoperative complications after breast reconstruction.

The findings of this study show that the pattern of complications for the studied methods differs significantly. The LD group has the highest rate of minor complications, especially seroma, but the lowest rate of complications requiring re-surgery. DIEP patients had the lowest rate of minor complications but highest rate of complications demanding re-surgery. This finding differs from a previous study by Thorarinsson et al. [8], in which, the highest incidence of overall complications was registered in DIEP reconstructions as well as complications demanding re-surgery. On the other hand, other studies indicate that general complication rates, other than seroma, of LD flaps are comparable to perforator-based free flaps such as DIEP [10]. Differences were also observed in implant reconstructions which, in our study, had equal amount both minor complications and complications requiring surgery, while in earlier studies implant reconstructions had less minor complications than complications requiring re-surgery [8]. These differences might indicate that study populations in the studies are different and complication rates may be influenced by individual practice differences in different centers. Some difference may be explained also by grading of complications and the accuracy in registration. We used Clavien–Dindo classification in which all events deviating from normal postoperative course were registered and counted for as complication. We were able to collect all data of all patients and procedures were performed in a one center by same surgeons.

There are many studies evaluating postoperative complications and comorbidities associated with breast reconstructions [11–18], but only few have assessed the predictive risk factors for postoperative complications. It is common that only one or two surgical techniques are compared to or the follow-up time is 30 days postoperatively [14, 19, 20]. It has been concluded that traditional 30-day readmission rates are not an adequate quality metric for breast reconstruction given the number of late postoperative readmissions in breast reconstructions [11, 18]. In order to describe reliable data concerning complications in breast reconstructive surgery, it is important to systematically analyze and compare complications from different reconstructive methods. In this study, we did a systematic registration of complications identified within 90 days of operation in autologous and alloplastic reconstruction techniques both in delayed and immediate procedures.

The complication rate was higher after immediate reconstruction than delayed reconstruction. This finding is in agreement with earlier studies [1, 21]. In this study, the difference was statistically significant in complications requiring re-surgery, while seroma and superficial infection rates appeared to be comparable between immediate and delayed reconstructions. The assessments of immediate reconstruction, however, do not distinguish between mastectomy—and reconstruction-related complications. Complication rates reported for immediate procedures actually describe outcomes for two operations, while complications in delayed reconstruction are attributable only to reconstructive procedure.

In this study, immediate and delayed reconstruction cohorts included different patient populations. The immediate group tended to have younger patients with lower BMI and less likely to have radiation therapy. One-third of patients with immediate reconstruction received postoperative radiation therapy. Previous investigators have reported that immediate reconstructions receiving radiotherapy are associated with higher complication rates than delayed reconstructions performed after radiation therapy [1, 8]. We refer all breast cancer cases in our multidisciplinary meeting and recommend immediate reconstruction for those patients who are less likely to receive postoperative radiation therapy. This is in line with many plastic surgeons who still recommend delaying reconstruction in this setting to avoid adverse effects of radiation on breast reconstructions [1]. On the other hand, 57% of patient with delayed reconstruction had received preoperative radiation therapy. It is well established that radiotherapy increases the risk of complications at the site of the reconstruction [8]. Of implant reconstructions, 29% received radiation therapy compared to 51% of LD and 57% of DIEP reconstructions. This agrees with the other study, in which patients reconstructed with DIEP and LD had significantly higher rates of preoperative radiation therapy [8]. The use of implants in irradiated patients is controversial. Irradiation has significant negative effects on the reconstructive outcome with implants [22]. These include poor cosmesis, capsular contracture, pain, exposure and implant removal. In this study, 18% of implant patients had re-surgery demanding complication compared to 15% in LDs and 20% in DIEPs. Despite improved radiation equipment, the cellular changes and injuries caused by radiation therapy are unavoidable. Irradiated chest wall skin is contracted and thus the creation of a ptotic, supple breast is a challenge. The surgeon must provide adequate skin and subcutaneous tissue from the flap and the scar tissue must be sufficiently released [23]. Irradiation can explain some of the complications in this study; however, in multivariate analysis radiation therapy was not among the variables independently associated with complications.

In our study, BMI > 30 was one of the variables independently associated with complications. In earlier studies, high BMI is widely known to be a risk factor for adverse postoperative events in all types of surgery [8, 24], also, in breast reconstructions [14, 25–27]. A potential etiology of increased risks of wound complications in obese may be related to increased cardiac workload, impaired diaphragmatic descent secondary to large volume of adiposity resulting in decreased oxygenated blood flow to tissues. Habitus-related decreased mobility also increases hygiene-related complications [19]. More obese individuals are seeking breast reconstruction, increasing the importance of identifying and understanding risks in these patients. It has been concluded, however, that obese patients should be counseled regarding their relative risks, but high BMI should not be considered an absolute contraindication for breast reconstruction [14, 26]. Being fully aware of the possible complications and explaining them to patients does not, however, make the complication rates lower. Obese patients should be advised to reduce their body weight, at least before delayed operations.

Variables independently associated with complications in this study included also a history of asthma or chronic obstructive pulmonary disease. This is in line with earlier studies [16, 18] with free flaps and mastectomy combined with implant or muscular flap reconstruction [17]. In those studies, a significant correlation was found between COPD/asthma and the need for revision surgery. Of other comorbidities and risk factors, hypertension and tobacco smoking have also been associated with morbidity, delayed wound healing and poorer surgical outcome [14]. It has been suggested that antihypertensive drugs, especially angiotensin receptor blockers, are associated with significantly higher rate of fat necrosis and significantly greater odds for development of overall perfusion-related complications in DIEP reconstructions [13]. In our study, 18% of study population had cardiovascular disease and 9% of patients were smokers. Smoking increases platelet aggregation, cutaneous vasoconstriction and tissue hypoxia [14]. All patients were instructed to stop smoking 4 weeks prior the operation. However, we cannot be sure that all have really done so. We do not use any laboratory tests to identify smokers. The amount of smokers was highest in implant and LD groups. It is possible that smokers could have been more actively chosen to get other than microvascular reconstruction. It is also possible that smoking can explain some of the complications although, in multivariate analysis, neither cardiovascular disease nor smoking was independent risk factor for complications. It is important, however, that tobacco smoking is ceased at least 4 weeks prior surgery and preexisting illness is evaluated carefully with a close multidisciplinary approach preoperatively.

The increase in life expectancy has increased the number of elderly patients who seek reconstruction. The rate of breast reconstruction following mastectomy is, however, lower among older women compared with younger [15], which is in agreement with our study. Several previous studies have indicated that age is an independent risk factor for complications [8]. Several factors contribute to an increased risk of developing complications in patients with increasing age. At the age of 65, cardiac output is only 70% of that at the age of 30 and renal function is reduced by 50 percent [14]. In our study, the most common age in reconstruction patients was 45–65 years, and only 7% of patients were older than 65 years. Age was not an independent risk factor for complications in our study, which is in agreement with a previous study by Masoomi et al. [19]. This finding might, however, be influenced by a small amount of older patients in our study population.

In this study, we registered complications within 90 days of operation in different breast reconstruction methods. However, the long-term performance of these breast reconstruction techniques would be different. The different amount of secondary corrections might be required after primary operation before a stable reconstructive construct is obtained. In future, we are planning to analyze also the long-term performance in our study population.

There are several limitations to this study similar to retrospective studies. We were, however, able to collect all the data of all patients, readmissions and procedures. Because of greater prevalence of delayed reconstruction (85%), our sample size of immediate reconstructions (15%) was relatively small, as was also the rate of implant reconstructions (6%). A larger number of implant reconstructions likely would have impacted our results. Despite our use of multivariate analyses, our findings may have been confounded by unknown clinical or demographic variables. Our findings may not be generalizable to all practice settings. Other practice settings in other countries or geographic regions or cultures may achieve different outcomes after breast reconstruction.

## Conclusions

Our study presents that risk stratification by preoperative factors would be feasible for postoperative complications after breast reconstruction. Four variables, including BMI > 30, asthma/COPD, immediate reconstruction and LD technique, were significantly associated with postoperative complications. In increasing trend of patients with increasingly medically complex problems undergoing breast reconstruction, tailoring of preventive measures to patients’ unique risk factors and careful consideration of the best timing of reconstruction is mandatory to prevent complications and costs after breast reconstruction procedures.
